# New principle for busbar protection based on the Euclidean distance algorithm

**DOI:** 10.1371/journal.pone.0219320

**Published:** 2019-07-24

**Authors:** Xingxing Dong, Qian Peng, Hao Wu, Zhengwei Chang, Yinggao Yue, Yi Zeng

**Affiliations:** 1 Automation and Information Engineering, Sichuan University of Science and Engineering, Zigong, China; 2 Electric Power Research Institute, State Grid Sichuan Electric Power Company, Chengdu, China; Newcastle University, UNITED KINGDOM

## Abstract

A new fast busbar protection algorithm based on the comparison of the similarity of back-wave waveforms is proposed in this paper. The S-transform is performed on the back-wave from each defected transmission line connected to the busbar, and the protection criterion is thus constructed by using the Euclidean distance to analyze the similarity of the back-waves, with the implementation of the S-transform between the transmission lines. When a fault occurs internally on the busbar, the Euclidean distance of the S-transformed back-wave between each associated transmission line is small, and there is a remarkable similarity between the waveform. When a fault occurs externally on the busbar, the Euclidean distance of the S-transformed backward traveling wave between the faulty line and the nonfaulty line is larger than that between the nonfaulty lines. The wave-forms of the faulty line and the nonfaulty line bear little similarity, while there is a striking similarity between the nonfaulty lines. Therefore, a protection criterion is established according to the ratio between the maximal similarity and the minimal similarity to discriminate the internal and external faults of the busbar zones. The simulation results show that the proposed busbar protection method can discriminate the internal and external faults of busbar zones in a sensitive and reliable way.

## Introduction

In high-voltage power grids, busbar faults not only cause large-scale power outages but also stable damage to the power system. Therefore, busbar protection plays a very important role. To quickly eliminate the fault and ensure the safety of the system, it is necessary to equip the relay protection device with high, fast, and sensitive reliability and selectivity [1–3].

Bus protection can be divided into power frequency protection and transient protection according to the operation principle. Current differential protection is the most widely used power frequency protection, but it may be misjudged because of CT (Current Transformer) error or CT saturation [4–6]. To solve the problem of the weak anti-CT saturation ability of traditional power frequency bus protection, a digital differential bus protection scheme based on the generalized alpha plane method was proposed in reference [7]. The algorithm maps a period of the CT secondary current signal to the alpha plane for fault area identification, which can effectively avoid the traditional protection problem of a lack of anti-saturation of the CT. However, in order to satisfy the reliability of the protection, the filtering process is added, which increases the time of the protection action. Reference [8] proposed a bus differential protection principle that achieves adaptive characteristics by using the alienation protection principle and the differential protection principle. Although the latter principle has a better performance than traditional differential protection, it increases the fault diagnosis time when CT saturation occurs. Even though the above literature solves the traditional differential protection’s problem of an insufficient CT saturation resistance, the speed of protection operation in a UHV/EHV(extra-high voltage and extra-high voltage) power grid is slightly insufficient.

Although traditional transient protection can achieve a high-speed operation, its stability still needs to be improved. A low impedance bus protection based on a wavelet transform has been proposed in the literature [9]. Although the 87BW function can improve the speed of bus protection and has a better CT saturation resistance, the performance of the protection algorithm has only been verified under the condition of a 50 dB signal-to-noise ratio. Although many simulations have been performed, the performance of the protection algorithm under the condition of a lower signal-to-noise ratio has not yet been verified. The 87BW function may also not work properly when the fault causes transient overdamping. Reference [10] defined the transient traveling wave power. A wavelet transform was then used to identify and compare the direction of the transient traveling wave power of each line. According to the direction characteristics of the traveling wave power of all lines during a fault, the internal and external faults of the buses can be distinguished. However, the principle is greatly affected by the initial small angle of the fault. In reference [11], a bus fault area identification method based on a polarity comparison of a superimposed current was proposed. However, strict filtering measures are needed to filter the fault transient high frequency signals, and the filtering delay reduces the operation speed of the protection. Reference [12] used the same magnitude and negative polarity of the measured impedance of each circuit in the bus area when a fault occurred, as well as the opposite polarity of the impedance of the fault line and the nonfault line in order to distinguish between a fault inside and outside of the bus area. However, only using the traveling wave head information results in the reliability of the criterion being insufficient. To improve the reliability of transient protection, reference [13] proposed an HHT transform of the traveling fault wave. The amplitude of the traveling fault wave was then selected for integration. Finally, the integral value was compared with the threshold value to judge the bus fault area. Reference [14] studied the characteristics of the voltage direction traveling wave of the connecting line when internal and external faults of the bus occurred, then integrated the corresponding forward and reverse traveling waves, constructing the bus fault identification criterion by using the ratio of the integral value. However, the protection performance was not analyzed or verified when the sampling value was lost. Reference [15] used an SVM and an S-transform to identify the fault area and achieved a good fault classification accuracy under the condition of system parameters diversification. Reference [16] proposed a bus protection scheme based on the Relevant Vector Machine (RVM), which reduced the relevant parameters and kernel functions in the calculation on the basis of traditional SVM. However, due to the mutation of the kernel function, the probability predictions of bus protection schemes based on SVM are not reliable.

To overcome the deficiency of traditional busbar protection against CT saturation and the contradiction between speed and reliability, and based on the theory of directional traveling waves and the correlation degree described in reference [17–18] combined with the application of an S-transform in a power system, this paper obtains the backward traveling wave after an S-transform of each related line of the busbar in a period of time after a fault. The Euclidean distance algorithm is used to identify the faults into and out of the busbar area. The new principle avoids the misjudgment caused by the loss of the traveling wave head in traditional traveling wave protection. Compared with the traditional power frequency relay protection, there is no problem of CT saturation and misjudgment, and the speed of the operation is faster. At the same time, using the information from 100 sampling points, the criterion can identify the fault area reliably, even when the initial angle of the fault is small. Compared with traditional traveling wave protection, it is more sensitive and reliable to use the current polarity or amplitude as the criterion, and an S-transform also plays a filtering role in processing the traveling wave signal to a certain extent, as well as having a certain anti-noise ability. The results of the theoretical analysis and experimental simulation show that the algorithm can identify the internal and external faults of the busbar both sensitively and reliably under various operating conditions.

## Analysis of the characteristics of a fault current traveling wave

### Basic fault branch detection theory of traveling fault waves

**Fig 1:**
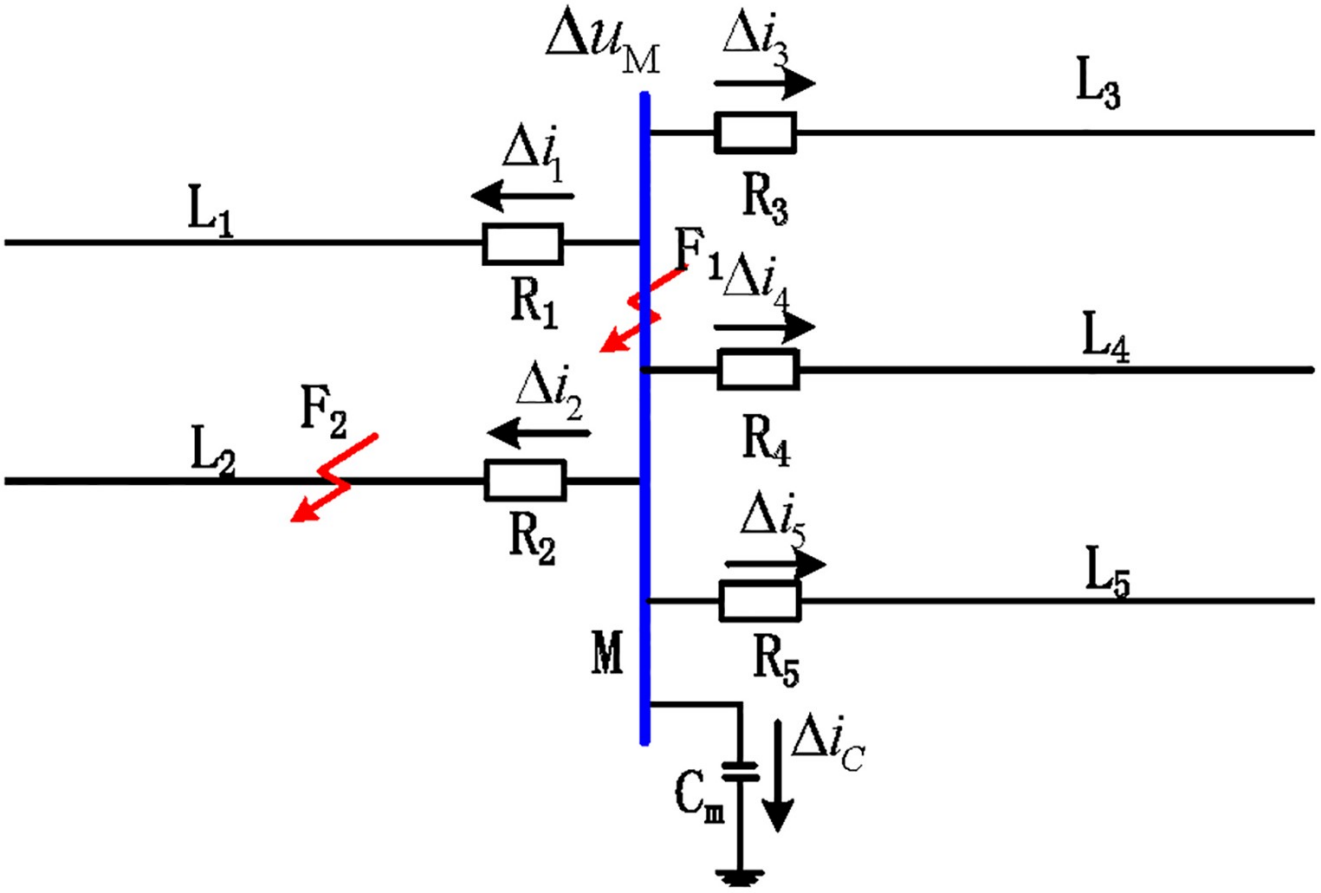
Sketch map of a 500 kV busbar system.

Fig 1 shows the busbar of the 500 kV substation, where L_1_-L_5_ are the five transmission lines connected to busbar M, and R_1_-R_5_ are the traveling wave protection units for the corresponding transmission lines installed near the line terminals that connected to the busbar. When a fault occurs at F_2_ on line L_2_, a travelling wave propagates from the fault point along the line to both sides, where reflection and refraction may occur due to wave impedance discontinuities. For any point on the transmission line at a distance of *x* to the fault, the transient voltage and current at this point can be derived [13]:

$$\left\{ \begin{array}{l} \Delta u(\text{x},\text{t})=\Delta u_{+}(\text{x}-\text{tv})+\Delta u_{-}(\text{x}+\text{tv})\\ \Delta i(\text{x},\text{t})=\Delta i_{+}(\text{x}-\text{tv})+\Delta i_{-}(\text{x}+\text{tv})\\ v=1/\sqrt{LC} \end{array}\right.$$ (1)

In the equation, t is the observation time; L and C are the inductance and capacitance per unit length of the transmission line; Δ*u*_+_(Δ*u*_−_) and Δ*i*_+_(Δ*i*_−_) are the voltage and current forward (backward) traveling wave propagating along the positive (opposite) direction of *x*, respectively.

According to the traveling wave propagation theory, the time when the initial traveling wave reaches busbar M is set as *t*_0_, and the time when the traveling wave is deflected and reflected and reaches busbar M for the second time is set as *t*_1_. Therefore, in the time period *t*_0_ ~ *t*_1_ the traveling fault waves obtained by the protection unit R_k_ (k = 1, 2, 3, 4, 5) of each transmission line connected to the busbar are called the initial voltage traveling wave and initial current traveling wave. Δ*u*_*M*_ is the initial voltage traveling wave of busbar M, and Δ*i*_*k*_(*k* = 1, 2, 3, 4, 5) is the current traveling wave measured from each line of busbar M. Z_c1_-Z_c5_ are the wave impedances of the associated transmission lines L_1_-L_5_ connected to the busbar, and the equivalent impedance of the busbar-to-ground stray capacitance is Z_cm_.

### Analysis of the fault current traveling wave propagation process

#### Characteristics of the current traveling wave when an internal fault occurs on the busbar

An analysis shows that the transient voltage and current at any point on the transmission line are superpositions of the forward and backward traveling waves, respectively. The current forward and backward traveling waves derived by Eq (1) are [13]:

{Δi+=12(Δi+Δuzc)Δi−=12(Δi−Δuzc) (2)

In the equations, Δ*u* and Δ*i* are the voltage and current fault component measured at point R on each line; and *Z*_*c*_ is the wave impedance of the transmission line.

**Fig 2:**
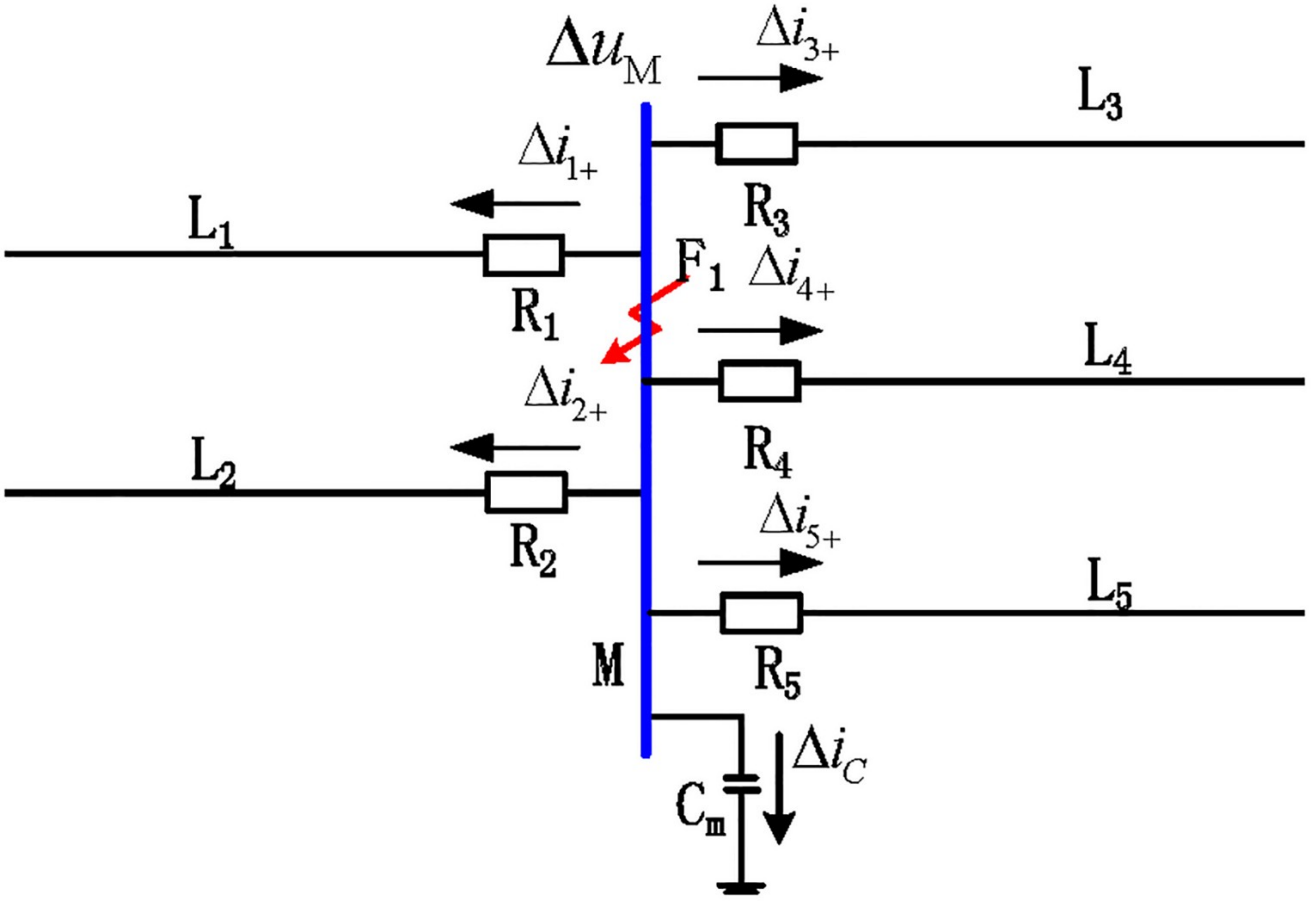
Propagation of the traveling fault wave when an internal fault occurs on the busbar.

According to the propagation characteristics of a traveling wave, the reflection and refraction of a traveling wave would occur at the fault point and on the busbar [13]. Referring to Fig 1, the positive direction of the traveling wave is defined as the transmission line at which the busbar points. When an internal busbar fault occurs, the traveling wave propagation mode is as shown in Fig 2. In the figure, Δ*i*_*n*+_(*n* = 1, 2, 3, 4, 5) represents the forward traveling wave of the nth transmission line.

When an internal fault occurs on the busbar, each outgoing line is a transmission line with evenly distributed parameter characteristics, and neither reflection nor refraction of wave impedance occurs on the transmission line. We assume that the length of the shortest line L among the associated transmission lines is *d*_min_. Therefore, in the time period [*t*_0_, *t*_0_ + 2*d*_min_/*v*], the initial forward traveling wave can be detected at R on each related transmission line, but there is no backward traveling wave formed by the reflection of the forward traveling wave.

#### Characteristics of the current traveling wave when an external fault occurs on the busbar

**Fig 3:**
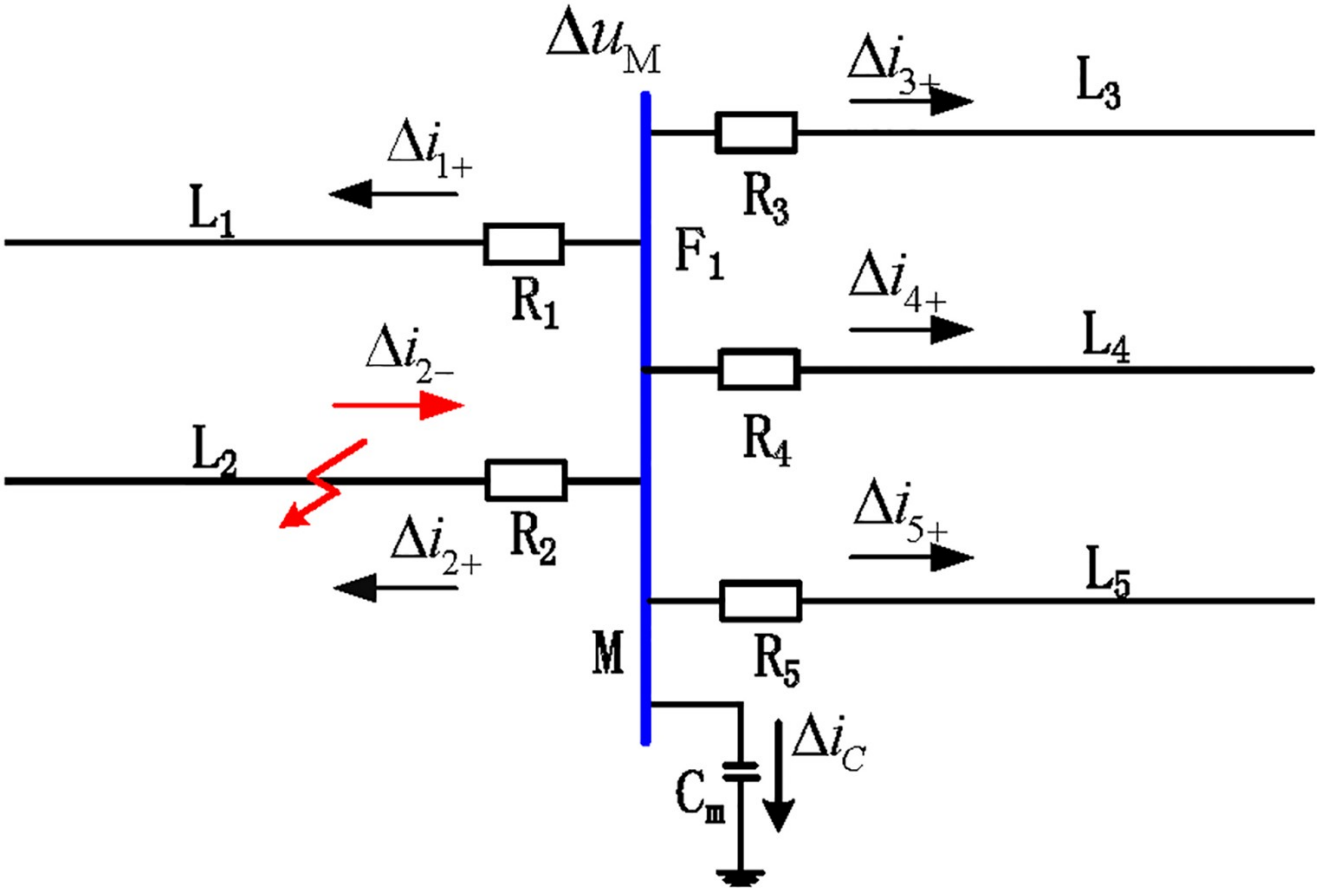
Propagation of the traveling fault wave when an external fault occurs on the busbar.

Fig 3 shows the propagation of the forward and backward traveling waves when L_2_ fails. In the figure, Δ*i*_*n*+_(*n* = 1, 2, 3, 4, 5) is the forward traveling wave of each associated line, and Δ*i*_2−_ is the backward traveling wave of L_2_. Since reflection and refraction of the backward traveling wave of L_2_ occur when reaching the busbar, the forward traveling wave Δ*i*_2+_ is formed by the reflection of the backward traveling wave. During the time period [*t*_0_, *t*_0_ + 2*d*_min_/*v*], the backward traveling waves can only be detected from fault transmission lines.

According to the analysis, when a fault occurs within the busbar zone, only forward traveling waves of each transmission line can be detected in the time period [*t*_0_, *t*_0_ + 2*d*_min_/*v*]. When a fault occurs outside the busbar zone, backward traveling waves can be detected from the fault line within the time period [*t*_0_, *t*_0_ + 2*d*_min_/*v*].

## Calculating the similarity of the backward travelling wave’s waveform based on the Euclidean distance

For a three-phase transmission system, there is coupling between the voltage and current of each phase. To eliminate the influence of coupling on the voltage and current, decoupling is generally performed by using phase transformations. In this paper, the phase-mode transformation is implemented by using a combined modulus method to reflect various fault types [17]:

$$\left\{ \begin{array}{l} \Delta u_{z}=4\Delta u_{\alpha }+\Delta u_{\beta }\\ \Delta i_{z}=4\Delta i_{\alpha }+\Delta i_{\beta } \end{array}\right.$$ (3)

This paper applies the method used in reference [18] to perform a discrete S-transform on the fault current traveling wave modulus after the implementation of the phase-mode transformation, and the Euclidean distances between the branch circuits are calculated by using the information of the initial current traveling wave.

### S-Transform theory

The S-transform is an extension of the principle of the wavelet transform and the short-time Fourier transform, which avoids the selection of a window function and makes up for the deficiencies of a fixed window width. At the same time, the characteristic quantity extracted by the S-transform is not susceptible to noise [13].

Assuming the continuous time signal is *h*(*t*), the continuous S-transform *S*(*τ*, *f*) of *h*(*t*) can be defined as follows:

$$S(\tau ,f)=\int_{-\infty }^{\infty }h(t)g(\tau -t,f)e^{-i2\pi ft}dt$$ (4)

$$g(\tau -t,f)=\frac{\left|f\right|}{\sqrt{2\pi }}e^{-\frac{{\left(\tau -t\right)}^{2}}{2\sigma^{2}}}$$ (5)

In the equations, *τ* is the parameter that controls the position of the Gaussian window on the time axis, *f* is the continuous frequency, *t* is time, *i* is an imaginary unit, *σ* = 1/|*f*| and *g*(*τ*-*t*, *f*) is Gaussian window, which is affected by the change of frequency.

If *h*[*kT*](*k* = 0, 1, 2 ⋯, *N* − 1) is the discrete time sequence obtained by sampling the signal *h*(*t*), T is the sampling interval, and N is the number of sampling points, then the discrete Fourier transform function of *h*[*kT*] is:

$$h[\frac{n}{NT}]=\frac{1}{N}\sum_{k=0}^{N-1}h[kT]e^{-j\frac{2\pi kn}{N}}$$ (6)

In the equation, *n* = 0, 1, ⋯, *N*−1.

Then, the discrete S-transform of the signal *h*(*t*) is:

$$S[kT,\frac{n}{NT}]=\sum_{r=0}^{N-1}h(\frac{r+n}{NT})e^{-\frac{2\pi^{2}r^{2}}{n^{2}}}e^{{}^{j\frac{2\pi rk}{N}}},n\neq 0$$ (7)

$$S[kT,0]=\frac{1}{N}\sum_{r=0}^{N-1}h(\frac{r}{NT}),n=0$$ (8)

The complex matrix from using an S-transform reflects the time domain and frequency domain characteristics of the signal, as well as the amplitude information and phase information of the traveling wave in the time domain.

### Similarity measure

#### Euclidean distance

Euclidean Space is a generalization of the two-dimensional and three-dimensional space studied by Euclid in mathematics. This so-called generalization converts the concept of distance and related concepts, such as length and angle made by Euclid, into an arbitrary number in a dimensional coordinate system [19]. The S-transformed waveform is a two-dimensional graph. Setting *x*_*i*_ and *y*_*i*_(*i* = 1,2 ⋯ *n*) as the continuous points in the two dimensional metric space of signal *x* and signal *y*, the Euclidean distance of *x* and *y* can be defined as follows [19]:

$$d\left(x,y\right)=\sqrt{\sum_{i=1}^{n}{\left(x_{i}-y_{i}\right)}^{2}}$$ (9)

In the equation, *x*_*i*_ and *y*_*i*_ are the *i*th sampling data points of signal *x* and signal y, respectively, and *n* is the total number of sampling points.

An analysis of Eq (9) shows that the higher the similarity between signal *x* and signal *y* is, the smaller the value of the Euclidean distance *d*(*x*,*y*) is. Conversely, the lower the similarity between signal *x* and signal *y* is, the larger the value of the Euclidean distance *d*(*x*,*y*) is.

Since the amplitude of the backward traveling wave waveform is relatively small, it is not possible to accurately identify the fault zone by simply comparing the Euclidean distances.

#### Similarity measure

To significantly reflect the difference in the waveform of the fault backward travelling wave of the related transmission lines via the Euclidean distance, the similarity is defined as:

$$sim\left(x,y\right)=\frac{1}{1+Kd\left(x,y\right)}$$ (10)

In this equation *K* is the reliability coefficient. Considering that the amplitude of the backward travelling wave and the difference in the Euclidean distances are relatively small, *K* is assigned a value 1000.

An analysis of Eqs (9) and (10) shows that the higher the similarity of the waveforms of signals *x* and *y*, the smaller the value of the Euclidean distance *d*(*x*, *y*), and the greater the similarity *sim*(*x*, *y*). Conversely, the larger the difference of the waveforms between signal *x* and *y*, the larger the value of the Euclidean distance *d*(*x*, *y*), and the smaller the similarity *sim*(*x*, *y*).

Take transmission line L_1_ as an example. (*sim*(*x*, *y*) = *sim*(*y*, *x*)) can be derived by calculating the similarity between the S-transformed waveform of the L_1_ backward travelling wave and the S-transformed waveform of the other four transmission lines related to the busbar within 0.5 ms after a fault occurs.

Thus, the similarity of the S-transformed backward travelling wave waveforms between L_2_-L_5_ and the other transmission lines are:

$$\{\begin{array}{c} \begin{array}{c} \begin{array}{c} {\text{L}}_{\text{2}}:sim\left(1,2\right);sim\left(2,3\right);sim\left(2,4\right);sim\left(2,5\right)\\ {\text{L}}_{\text{3}}:sim\left(1,3\right);sim\left(2,3\right);sim\left(3,4\right);sim\left(3,5\right) \end{array}\\ {\text{L}}_{\text{4}}:sim\left(1,4\right);sim\left(2,4\right);sim\left(3,4\right);sim\left(4,5\right) \end{array}\\ {\text{L}}_{\text{5}}:sim\left(1,5\right);sim\left(2,5\right);sim\left(3,5\right);sim\left(4,5\right) \end{array}$$ (11)

### Analysis of the backward traveling wave

#### Current backward traveling wave when an internal fault occurs on the busbar

**Fig 4:**
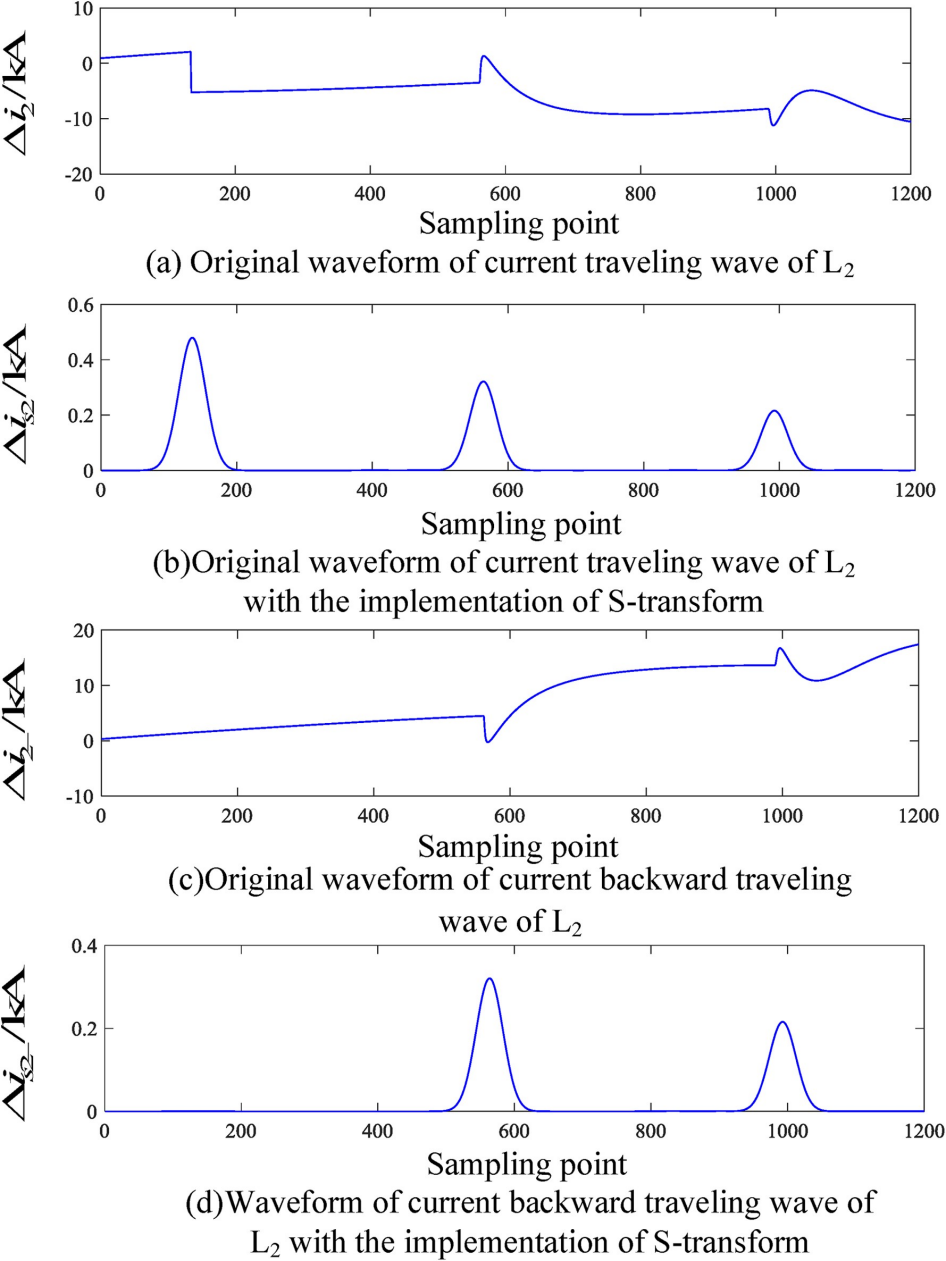
Corresponding traveling wave waveforms of L_2_ when a fault occurs on busbar M.

**Fig 5:**
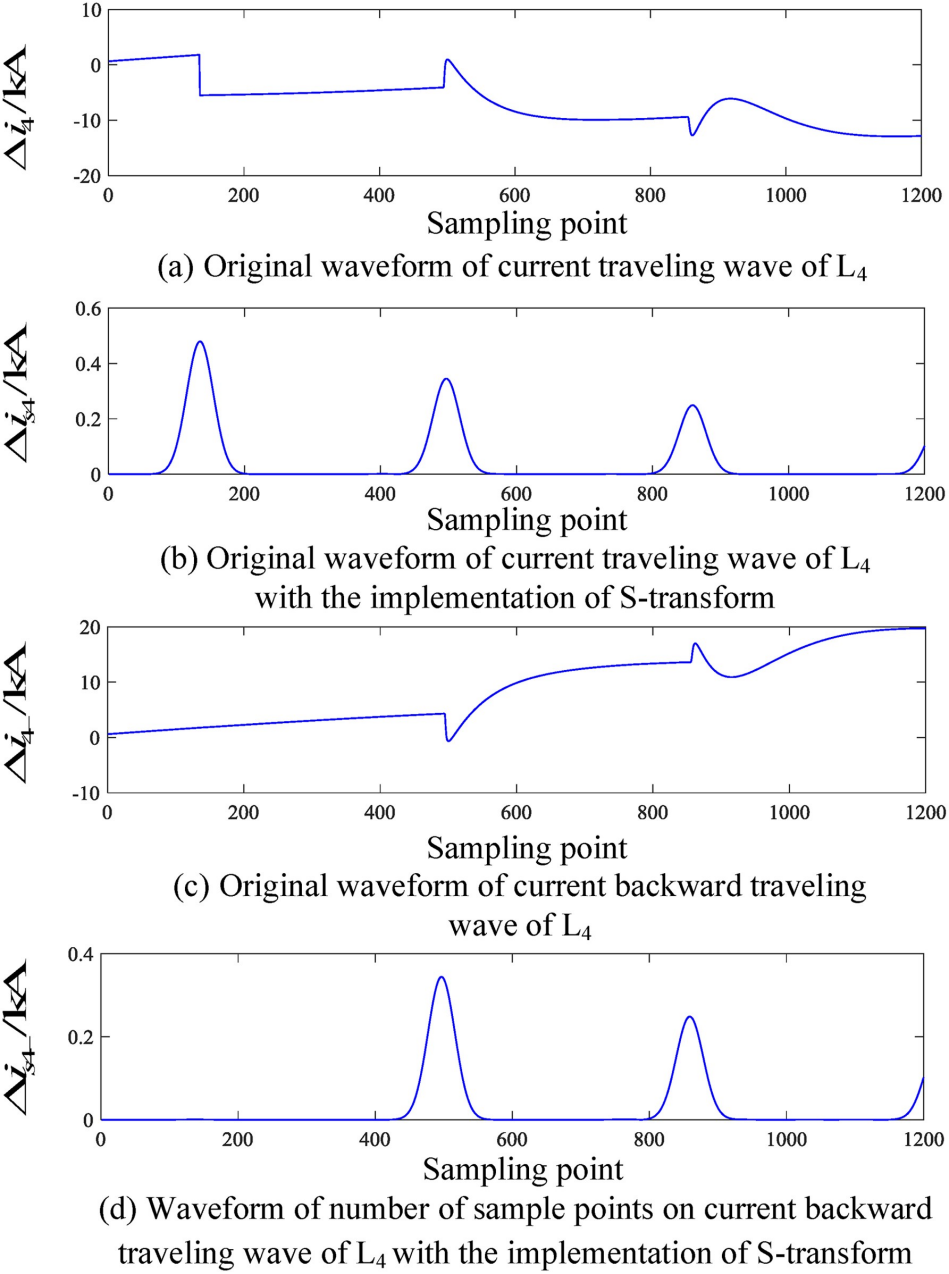
Corresponding traveling wave waveforms of L_4_ when a fault occurs on busbar M.

When an internal fault occurs in the busbar, the internal fault data obtained by the PSCAD(Power Systems Computer Aided Design) simulation is put into MATLAB(Matrix Laboratory) for the S-transformation simulation, and the corresponding traveling wave waveform is obtained. The corresponding current traveling wave waveforms of the related transmission lines (taking L_2_ and L_4_ as examples) are shown in Figs 4 and 5, respectively. In Figs 4 and 5, Δ*i*_*n*_(*n* = 1, 2, 3, 4, 5) denotes the corresponding original traveling wave, Δ*i*_*n*−_(*n* = 1, 2, 3, 4, 5) and denotes the corresponding backward traveling wave.

It can be seen from the analysis of Figs 4 and 5 that when a fault occurs within the busbar zone in the time period [*t*_0_, *t*_0_ + 2*d*_min_/*v*], there is hardly any backward traveling wave detected on the transmission line when the initial fault current travelling wave is detected from each related transmission line.

#### Current backward traveling wave when an external fault occurs on the busbar

**Fig 6:**
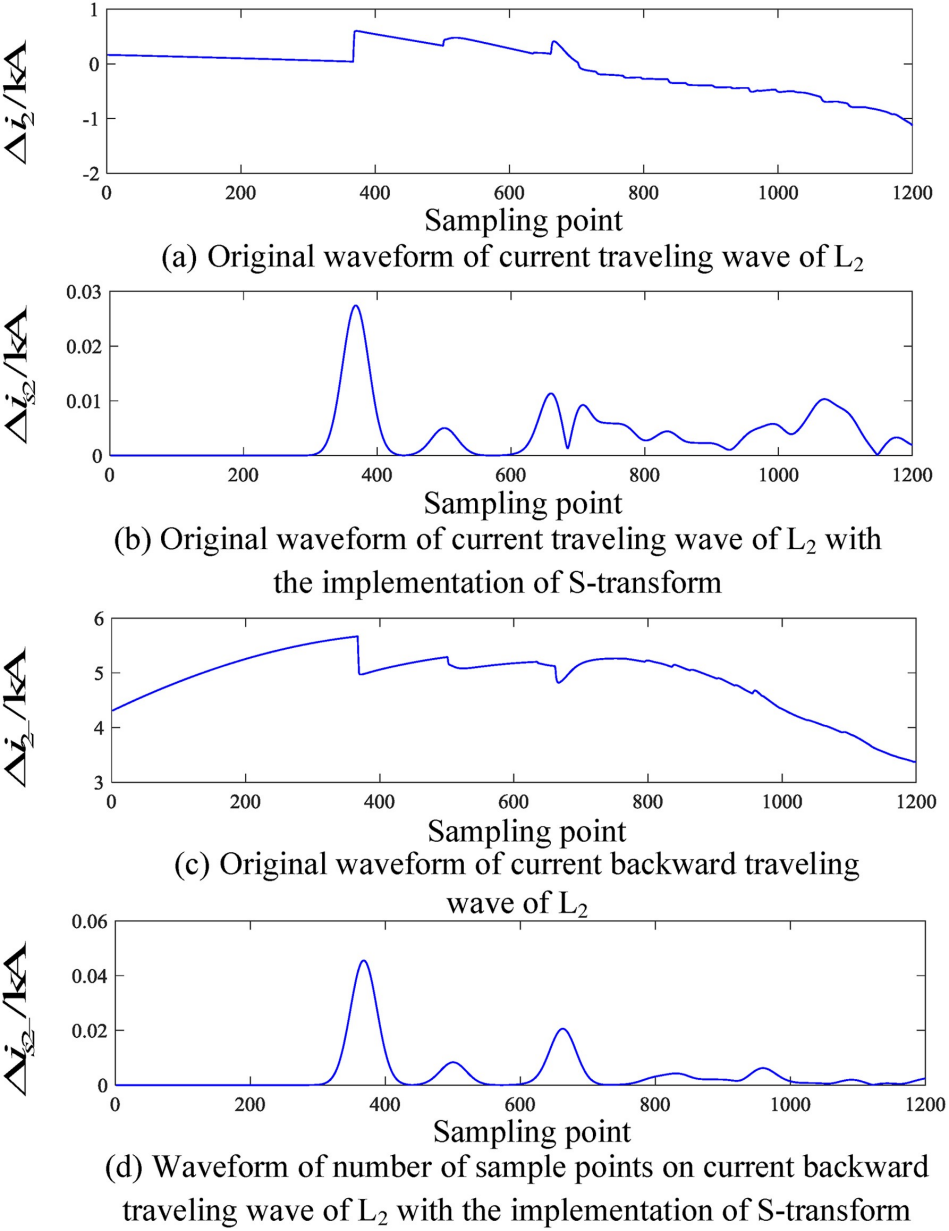
Corresponding traveling wave waveforms of L_2_ when a fault occurs on L_2_.

**Fig 7:**
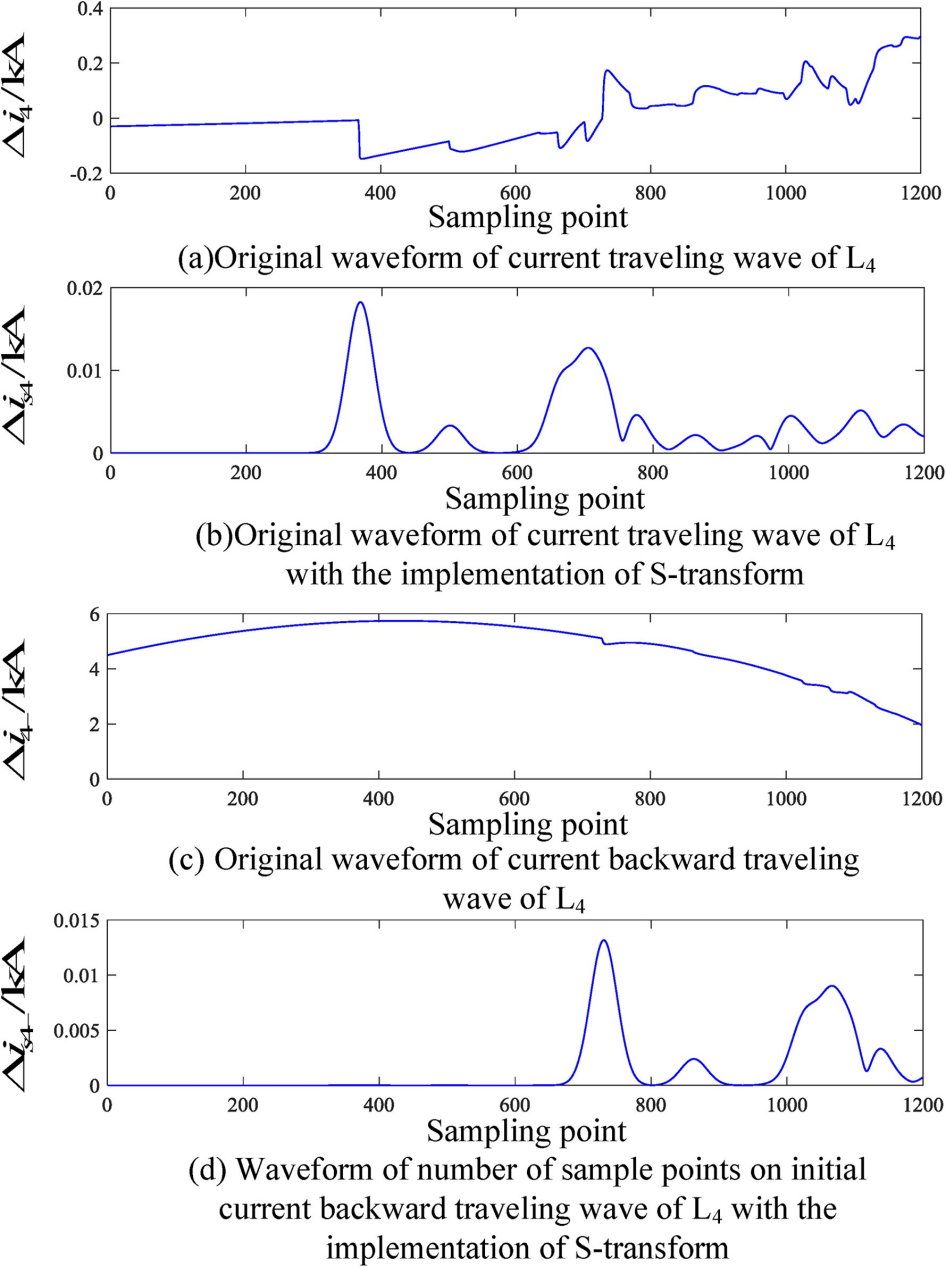
Corresponding traveling wave waveforms of L_4_ when a fault occurs on L_4_.

When a fault occurs on L_2_, the corresponding traveling wave waveform is obtained by employing an S-transform simulation. The corresponding traveling wave waveform of the fault line (taking L_2_ as an example) and the nonfault line (taking L_4_ as an example) are shown in Figs 6 and 7, respectively.

Similarly, Figs 6 and 7 show that when an external fault occurs, the faulted transmission line’s initial traveling wave and the faulted backward traveling wave appear simultaneously during the time period [*t*_0_, *t*_0_ + 2*d*_min_/*v*], and the backward traveling wave can be detected. For the nonfaulty transmission line, during the time period [*t*_0_, *t*_0_ + 2*d*_min_/*v*], the backward traveling wave is essentially not detected.

Based on the above analysis, in order to ensure that only the backward traveling wave or the forward traveling wave exist in the time period [*t*_0_, *t*_0_ + 2*d*_min_/*v*] of each associated transmission line of the busbar after a failure occurs, an appropriate time window needs to be selected. Since the total length of the associated transmission line of the 500 kV high voltage busbar system is generally above 100 km, this time 2*d*_min_/*v* is greater than 0.6 ms. Therefore, this paper selects 0.5 ms after the fault as the time window length, which is 100 sampling data points for the calculation basis.

## Busbar protection based on the similarity of the backward traveling wave waveforms

### Protection criteria

An analysis of Figs 4 to 7 shows that when an internal fault occurs in the busbar within time period [*t*_0_, *t*_0_ + 2*d*_min_/*v*], the waveforms of the corresponding travelling wave of the related transmission line are essentially the same, and the backward traveling wave can barely be detected. When an external fault occurs outside the busbar within time period [*t*_0_, *t*_0_ + 2*d*_min_/*v*], the backward traveling wave can be detected from the fault line and cannot be detected from the nonfault line. Therefore, an S-transform is deployed on the backward traveling wave using the characteristics of a backward traveling wave. Based on the transformed single-frequency backward traveling wave, the Euclidean distance between the related transmission lines can be derived and a protection criterion is thus established.

Taking the system of Fig 1 as an example, the Euclidean distances between the S-transformed backward traveling wave of the transmission lines connected to the busbar are *d*(*x*, *y*) and (*d*(*x*, *y*) = *d*(*y*, *x*)), and the similarity is *sim*(*x*, *y*) (*x* = 1, 2, 3, 4; *y* = 1, 2, 3, 4, 5; *x* ≠ *y*).

Eq (12) is defined according to Eq (11):

$$\{\begin{array}{c} M_{\text{s}}=\text{max}\left[sim\left(i,n\right)\right]\\ N_{\text{s}}=\text{min}\left[sim\left(i,n\right)\right] \end{array}$$ (12)

In this equation, when an internal fault occurs in the busbar, *i* = 1, 2, 3, 4, 5; *n* = 1, 2, 3, 4, 5(*i* ≠ *n*). When an external fault occurs on the busbar, the similarity between the fault line and the nonfault line is *sim*(*x*, *y*) ≈ 0, and the similarity between the nonfault lines is *sim*(*x*, *y*) ≈ 1, namely, *M*_s_ ≈ 1; *N*_s_ ≈ 0.

Based upon the above analysis, the following is defined:

$$\lambda =\frac{M_{\text{s}}}{N_{\text{s}}}$$ (13)

When an internal fault occurs in the busbar, *λ* ≈ 1, and when an external fault occurs on the busbar, *λ* ≫ 1. Based on the analysis above, a protection criterion can be established:

$$\lambda >K_{set}$$ (14)

In the equation, *K*_*set*_ = 5 is the threshold value.

### Analysis of the threshold value

Ideally, when an internal fault occurs in a busbar only forward traveling waves exist in the related transmission lines within time period [*t*_0_, *t*_0_ + 2*d*_min_/*v*], and there is no backward traveling wave. The detected amplitude of the backward travelling wave is approximately 0, the Euclidean distances between the related transmission lines are the same, and the similarity is essentially the same at approximately 1. When an external fault occurs on the busbar, the backward traveling wave can only be detected from the fault line within the time period [*t*_0_, *t*_0_ + 2*d*_min_/*v*] and the Euclidean distance between the fault line and the nonfault line is obviously larger than the Euclidean distance between the nonfault lines. Thus, the similarity coefficient between the fault line and the nonfault line is relatively small and approximately 0, and the similarity coefficient between the nonfault lines is relatively large and essentially equal to 1.

When an internal fault occurs in the busbar, no backward travelling wave can be detected within the time period [*t*_0_, *t*_0_ + 2*d*_min_/*v*]. At this time, *M*_s_ = max[*sim*(*i*, *n*)] ≈ 1, *N*_s_ = max[*sim*(*i*, *n*)] ≈ 1, and P = *M*_s_/*N*_s_ ≈ 1. When an external fault occurs on the busbar, a loss of sampling points of the fault line would result in a decrease in *sim*. However, because there is no large area loss, *M*_s_ ≈ 1, *N*_s_ < 1, and *P* = *M*_s_/*N*_s_ > 1. An analysis of Eq (11) shows that because K = 1000 and the backward travelling wave peak phasor of line model in this paper is more than 0.02 kA when the fault occurs, even if the Euclidean distance *d*(2, *n*)(*n* = 1, 3, 4, 5) between the transmission lines related to L_2_ only considers the peak point amplitude, then min[*sim*(*i*, *n*)] ≈ 1/3, and $\lambda =M_{\text{s}}/N_{\text{s}}=1/\frac{1}{3}=3$. At the same time, considering the influence of noise, through the simulation verification the threshold value is selected as *K*_*set*_ = 5 in this paper. The correctness of the threshold value selection will be verified with a large number of simulations.

In summary, considering the influence of the calculation error, noise interference, etc., the threshold value *C*_*set*_ is set as 3.5. According to the simulation results, the protection criterion has a high sensitivity and reliability.

### Selection of single-frequency of an S-transformed traveling wave

The value of a single frequency of the traveling wave using the S-transform determines the corresponding value of the amplitude of each sampling point, and plays an important role in the reliability, sensitivity and threshold value selection of the protection criterion. To clearly reflect the fault characteristics through the waveform of an S-transformed traveling wave, it is necessary to determine the most suitable traveling wave single frequency.

**Fig 8:**
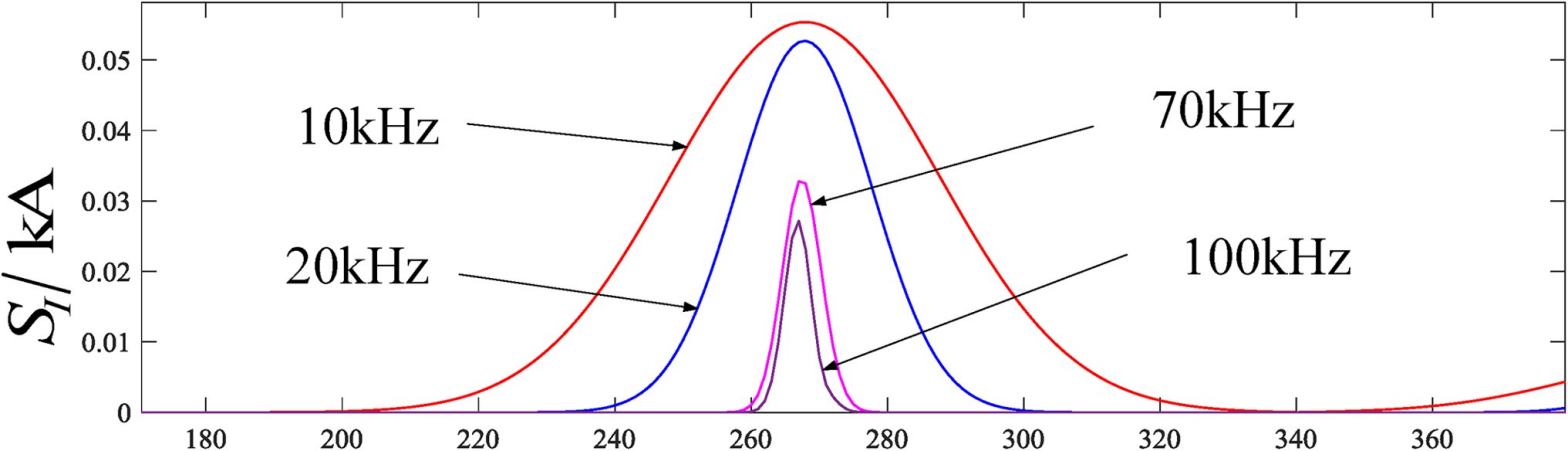
Partial current waveform at a single frequency with the implementation of an S-transform.

Taking the simulation model built in this paper as an example, Fig 8 is a current waveform corresponding to different single-frequency traveling waves by using an S-transform under the same fault conditions when the sampling frequency is 200 kHz, wherein the S-transformed single frequency of the waveforms from external to internal is 10 kHz to 100 kHz, respectively. It can be seen from the waveform of Fig 8 that the traveling wave single frequency ranges from 10 kHz to 100 kHz, the peak current gradually decreases with an increase in the frequency, and the waveform gradually narrows, thus fewer points can be sampled. Therefore, it can be seen that within the range of 10 kHz to 100 kHz, the current peak is the largest at a single frequency of 10 kHz with the implementation of an S-transform and more effective points can be sampled. Therefore, this paper selects the 10 kHz S-transformed single-frequency for the fault simulation.

However, it should be noted that since the parameters of different power system models vary, the difference in the length of the data window and the traveling wave single frequency that best reflects its fault characteristics may change. Therefore, different systems need to use a simulation analysis to determine the suitable traveling wave single frequencies, which are not necessarily 10 kHz.

### Protection algorithm flow

An S-transform is used on the reverse traveling wave measured from each transmission line connected to the busbar, and an S-transformed single-frequency fault current traveling wave corresponding to 10 kHz is selected to calculate the similarity between the backward traveling waves.

**Fig 9:**
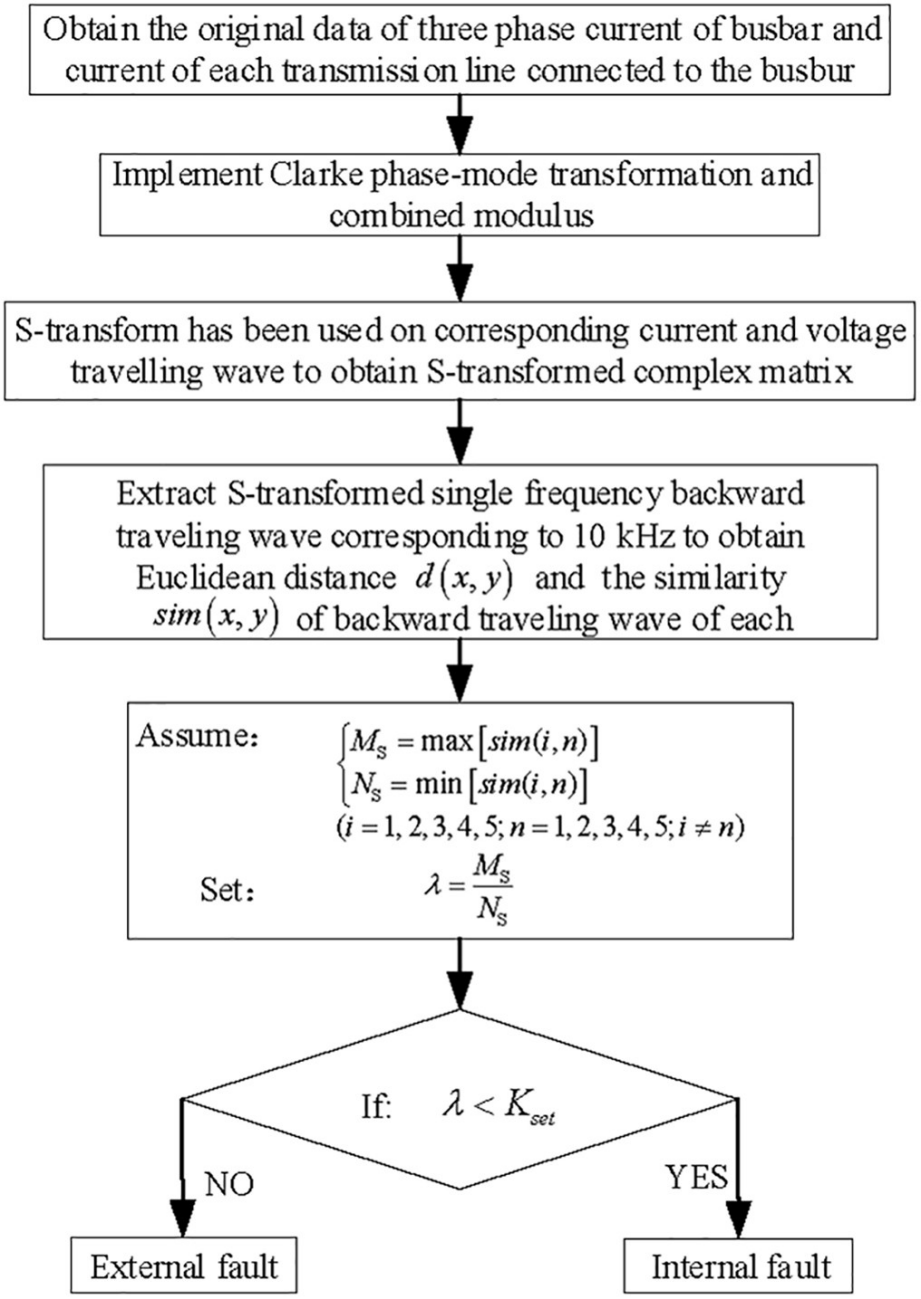
Busbar protection algorithm flow based on the current backward traveling wave.

The relationship between the ratio of the maximum similarity and the minimum similarity as well as the threshold value is analyzed to identify the internal and external faults that occurred on the busbar. The protection algorithm flow is shown in Fig 9.

## Simulation study

To test the rationality of the busbar protection algorithm for the comparison of the backward traveling wave waveform similarity, a simulation model of a 500 kV busbar system shown in Fig 1 is established using PSCAD/EMTDC(Electromagnetic Transients including DC) electromagnetic transient simulation software. The busbar adopts an LGJQT-1400 special light steel core aluminum stranded wire [20], and the line uses the structural parameters of the 500 kV transmission line between Pingdingshan to Wuhan of the Central China Grid [21] (the wire uses a LGJQ-300×4 four-split wire, and the single wire resistance is 0.108 Ω/km). Among them, the stray capacitance of the busbar is set as *C*_m_ = 0.01*μF*, the sampling frequency is 200 kHz, L_1_ = L_3_ = 250 km, L_2_ = 320 km, L_4_ = 270 km, and L_5_ = 300 km.

The S-transformed initial backward traveling wave signal corresponding to 10 kHz is selected, and the similarity is calculated by using the Euclidean distance.

### Busbar internal fault

We assume that an AB phase-to-ground short circuit occurs on busbar M (at fault point F_1_), and the initial fault angle is 45°. According to the analysis above, the similarity of the single-frequency backward traveling wave at the corresponding time of the initial traveling wave head can be calculated, and the following equations can be derived: *sim*(*i*, *n*) = 1.00(*i* = 1, 2, 3, 4, 5; *n* = 1, 2, 3, 4, 5; *i* ≠ *n*).

$$\begin{array}{ll} \{\begin{array}{c} M_{\text{s}}=\text{max}\left[sim\left(i,n\right)\right]=1.00\\ N_{\text{s}}=\text{min}\left[sim\left(i,n\right)\right]=1.00 \end{array}; & \lambda =\frac{M_{\text{s}}}{N_{\text{s}}}=1.00<K_{set} \end{array}$$

The criterion in Eq (14) is satisfied, thus it can be concluded that an internal fault occurs in the busbar and the protection operates.

To fully verify the effectiveness of the algorithm, several simulation experiments are conducted in this paper. Tables 1 to 3 show the simulation verification results of the protection under different conditions when an internal fault occurs on the busbar.

**Table 1:** Test results of the protection algorithm in the case of different fault inception angles when an internal fault occurs.

Fault location Fault type	Transitional resistance /(Ω)	Fault inception angle/(°)	M_s_	N_s_	λ	Test result
C phase-to-ground fault occurs on busbar M	300	2	1.00	1.00	1.00	Internal
		15	1.00	1.00	1.00	Internal
		30	1.00	1.00	1.00	Internal
		90	1.00	1.00	1.00	Internal
		120	1.00	1.00	1.00	Internal
AB phase-to-ground fault occurs on busbar M	800	2	1.00	1.00	1.00	Internal
		15	1.00	1.00	1.00	Internal
		30	1.00	1.00	1.00	Internal
		90	1.00	1.00	1.00	Internal
		120	1.00	1.00	1.00	Internal

**Table 2:** Test results of the protection algorithm in the case of different transitional resistances when an internal fault occurs.

Fault location	Transitional resistance/Ω	M_s_	N_s_	λ	Test result
AB phase-to-ground fault occurs on busbar M (Fault inception angle is 45°)	0	1.00	1.00	1.00	Internal
	200	1.00	1.00	1.00	Internal
	500	1.00	1.00	1.00	Internal
	800	1.00	1.00	1.00	Internal

**Table 3:** Test results of the protection algorithm in the case of different fault types when an internal fault occurs in the busbar.

Fault location	Fault type	M_s_	N_s_	λ	Test result
A fault occurs on busbar M; Transitional resistance is 200Ω (Fault inception angle is 60°)	AG	1.00	1.00	1.00	Internal
	BCG	1.00	1.00	1.00	Internal
	AB	1.00	1.00	1.00	Internal
	ABC	1.00	1.00	1.00	Internal

In Table 1 we assume that a C phase-to-ground fault and an AB phase-to-ground fault occur on busbar M in order to test the sensitivity of the algorithm in the case of different fault inception angles. The analysis shows that when the initial angle of the fault changes, the similarity *sim* of each related transmission line is essentially not susceptible to the fault inception angle, and the ratio λ of each line is smaller than the threshold value *K*_*set*_. That is, the protection algorithm can accurately identify the internal fault that occurred in the busbar zone at different fault inception angles.

To verify the performance of the protection algorithm in the case of different fault transition resistances, in Table 2 we assume that an A phase-to-ground fault occurs on busbar M in the case of different transitional resistances. It can be seen from the simulation results that the similarity *sim* essentially remains unchanged in the case of different transitional resistances, and the ratio *λ* can satisfy the criterion in Eq (14). That is, the protection criterion can correctly identify the busbar fault in the case of different fault transitional resistances.

Table 3 verifies the protection performance of the algorithm in the case of different fault types. It can be seen from the simulation results that the similarity *sim* is not susceptible to a transitional resistance, and the ratio *λ* can satisfy the criterion in Eq (14). That is, the protection criterion can identify the busbar fault in the case of different fault types.

According to the busbar internal fault simulation results, it can be concluded that the equation *sim*(*i*, *n*) = 1.00(*i* = 1, 2, 3, 4, 5; *n* = 1, 2, 3, 4, 5; *i* ≠ *n*) is satisfied no matter what types of faults occur. Considering the interference factors in actual operation, the similarity may be slightly fluctuated, so the threshold value *K*_*set*_ is set at 5 to ensure the sensitivity and reliability of the protection criterion.

In summary, when a fault occurs in a busbar zone, there is only forward traveling wave in the time period [*t*_0_, *t*_0_ + 2*d*_min_/*v*], with no backward traveling wave existing. The detected amplitude of the backward traveling wave is approximately 0, the Euclidean distance is essentially equal to 0 and the similarity is approximately 1. That is, when a fault occurs within the busbar zone, the algorithm satisfies the criterion in the case of different fault inception angles, different transitional resistances and different fault types. The simulation data is consistent with the theoretical analysis results, and the protection can sensitively identify the internal faults and operates in a reliable way.

### External faults occurring outside the busbar zone

Assume that a C phase-to-ground fault occurs at F_2_ on L_2_ at a distance of 100 km from busbar M. The initial fault angle is 90° and the transitional resistance is 200 Ω. It can be derived that:

$$\begin{array}{cc} \begin{array}{c} \{\begin{array}{c} M_{\text{s}}=\text{max}\left[sim\left(i,n\right)\right]=1.00\\ N_{\text{s}}=\text{min}\left[sim\left(i,n\right)\right]=0.0038 \end{array}\\ \left(i=1,2,3,4,5;n=1,2,3,4,5;i\neq n\right) \end{array}; & \lambda =\frac{M_{\text{s}}}{N_{\text{s}}}=\frac{1.00}{0.0038}\approx 263.16>K_{set} \end{array}$$

The criterion in Eq (14) is not satisfied, thus we can arrive at the conclusion that an external fault occurs outside the busbar protection zone.

In Table 4, L_2_ and L_4_ are chosen to conduct simulation experiment in the case of different fault inception angles. The simulation results show that the maximum similarity is approximately 1, the similarity ratios *λ* are greater than the threshold value *K*_*set*_, and the simulation data does not satisfy the criterion in Eq (14). That is, the protection algorithm can sensitively and reliably identify the external faults in the case of different fault inception angles.

**Table 4:** Test results of the protection algorithm in the case of different fault inception angles when an external fault occurs outside the busbar zone.

Fault location Fault type	Fault inception angle /(°)	M_s_	N_s_	λ	Test result
B phase-to-ground fault occurs on L_2_ at a distance of 80km from busbar M; transitional resistance is 100Ω	2	1.00	0.0032	312.50	External
	15	1.00	0.002	500.00	External
	30	1.00	0.0028	357.14	External
	90	1.00	0.0053	188.68	External
	120	1.00	0.0042	238.10	External
A phase-to-ground fault occurs on L_4_ at a distance of 50km from busbar M; transitional resistance is 150Ω	2	1.00	0.0035	285.71	External
	15	1.00	0.0038	263.16	External
	30	1.00	0.002	500.00	External
	90	1.00	0.01	1000.00	External
	120	1.00	0.0035	285.71	External

Table 5 verifies the impact of different fault transitional resistances on the protection algorithm. A C phase-to-ground short circuit fault that occurs on transmission line L_2_ at a distance of 300 km from busbar M and an AB phase-to-ground short circuit fault that occurs on L_4_ at a distance of 260 km from busbar M are set. The simulation results show that when the transitional resistance changes the maximum similarity is approximately 1, the minimum similarity is essentially 0, and the similarity ratio *λ* is greater than the threshold value *K*_*set*_. The criterion is not satisfied, so the algorithm is not susceptible to the change in the transitional resistance, therefore it can accurately identify an external fault outside the busbar zone.

**Table 5:** Test results of the protection algorithm in the case of different transitional resistances when an external fault occurs outside the busbar zone.

Fault location Fault type	Transitional resistances /(Ω)	M_s_	N_s_	λ	Test result
C phase-to-ground fault occurs on L_2_ at a distance of 100km from busbar M; fault inception angle is 90°	0	1.00	0.002	500.00	External
	200	1.00	0.0038	263.16	External
	500	1.00	0.0063	158.73	External
	800	1.00	0.0035	285.71	External
AB phase-to-ground fault occurs on L_4_ at a distance of 260 km from busbar M; fault initial angle is 60°	0	1.00	0.0005	2000.00	External
	200	1.00	0.0005	2000.00	External
	500	1.00	0.0005	2000.00	External
	800	1.00	0.0005	2000.00	External

To verify the impact of different fault types on the protection criterion, L_2_ and L_4_ are chosen to perform a simulation experiment in the case of different fault types. The simulation results are shown in Table 6. The analysis shows that the similarity between the nonfault lines is essentially equal to 1, the similarity between the fault lines is approximately 0, and the similarity ratio *λ* ≫ *K*_*set*_. The simulation data does not meet the criterion requirements; thus we can arrive at the conclusion that an external fault occurs outside the busbar zone. That is, the protection algorithm is not susceptible to changes in fault types.

**Table 6:** Test results of the protection algorithm in the case of different fault locations and fault types when an external fault occurs outside the busbar zone.

Fault location	Fault type	M_s_	N_s_	λ	Test result
A fault occurs on L_2_ at a distance of 20km from busbar M; transitional resistance is 80Ω; fault inception angle is 30°	AG	1.00	0.0015	666.67	External
	ABG	1.00	0.0005	2000.00	External
	BC	1.00	0.0018	555.56	External
	ABC	1.00	0.001	1000.00	External
A fault occurs on L_4_ at a distance of 120km from busbar M; transitional resistance is 150Ω; fault inception angle is 60°	AG	1.00	0.0013	769.23	External
	ABG	1.00	0.0005	2000.00	External
	BC	1.00	0.0025	400.00	External
	ABC	1.00	0.0005	2000.00	External

Because no backward traveling wave can be detected on the associated transmission line in the time period [*t*_0_, *t*_0_ + 2*d*_min_/*v*] when an internal fault occurs, the length of the shortest associated transmission line set by the busbar simulation model established in this paper is 250 km, so under the given 0.5 ms data window calculation condition, when an internal fault occurs no backward traveling wave can be detected from each busbar protection unit, and only the influence of the different fault distances on the protection criterion need to be considered when an external fault occurs. Table 7 shows the simulation analysis of faults occurring on L_2_ at different distances. The simulation results are as follows:

**Table 7:** Protection algorithm test results when different fault distances occur outside the busbar protection zone.

Fault type	Fault distance/(km)	M_s_	N_s_	λ	Test result
AB phase-to-ground fault occurs on L_2_ at different distances from busbar M, and the transitional resistance is 100Ω; fault Initial angle is 45°	0.2	3.000	0.0005	3.000	External
	0.5	3.000	0.0005	3.000	External
	1	3.000	0.0006	3.000	External
	30	3.000	0.001	3.000	External
	80	3.000	0.001	3.000	External
	180	3.000	0.002	3.000	External
	240	3.000	0.002	3.000	External
	310	3.000	0.002	3.000	External

An analysis of the simulation data in Table 7 shows that the protection criteria are not affected under various distances of external faults, and the fault zone can be accurately identified.

According to the simulation data of Tables 1–7, it can be seen that when an internal fault occurs in the busbar, the similarity ratios *λ* of each related transmission line are greater than the threshold value *K*_*set*_; when an external fault occurs outside the busbar protected zone, the similarity ratio *λ* of each transmission line is smaller than the threshold value *K*_*set*_.

This means that the algorithm can accurately identify the fault zone in the case of different fault inception angles, different transitional resistances and different fault types, and the protection can operate in a functionally reliable way. The test results of the simulation experiment are consistent with theoretical analysis.

## On the performance of the protection criterion

### Impact of noise on the protection criterion

**Fig 10:**
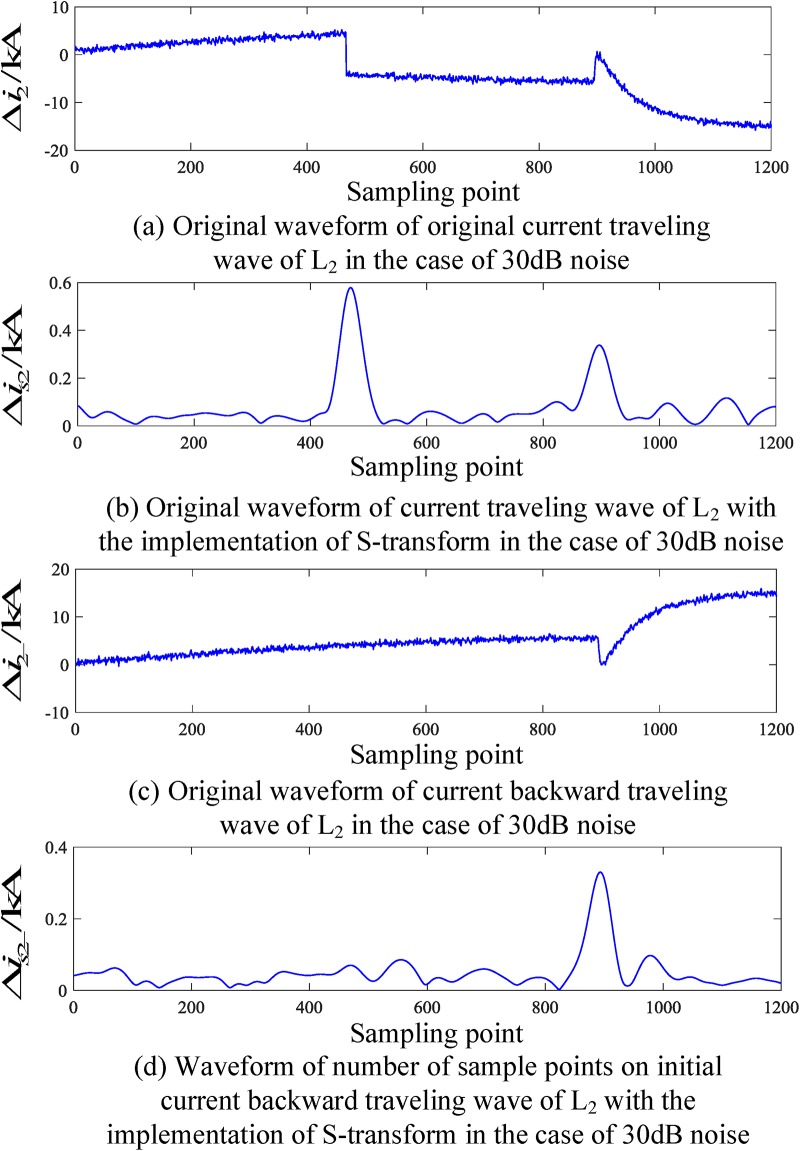
Corresponding current waveform of L_2_ in the presence of 30 dB noise when an internal fault occurs in the busbar.

**Fig 11:**
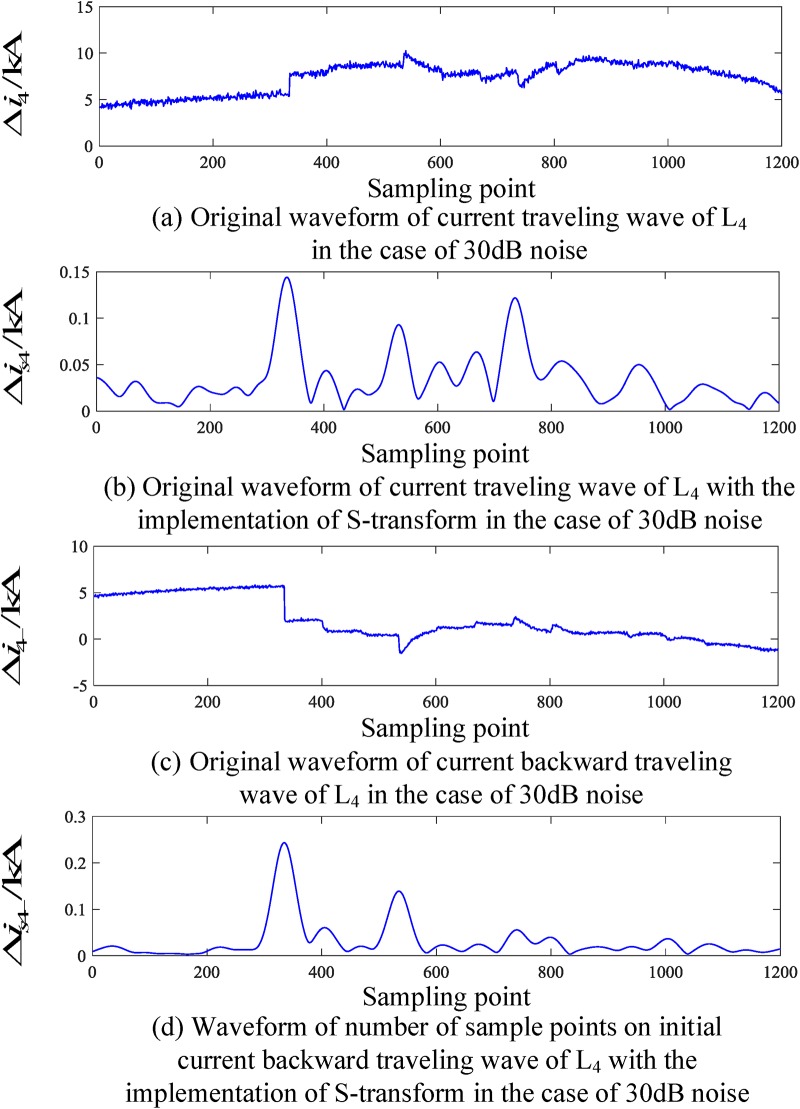
Corresponding current waveforms of L_4_ in the presence of 30 dB noise when a fault occurs on the transmission line.

To verify the reliability of the algorithm under the influence of noise, a simulation verification has been provided. The simulation condition is to add noise signals to the voltage signals of the busbar and current signals to the transmission lines connected to busbar, where the signal-to-noise ratio (SNRs) is 30 dB-70 dB. Figs 10 and 11 show the waveforms of the voltage and current traveling waves when an internal fault occur in the busbar, as well as the waveforms of the voltage and current traveling waves when an external fault occurs outside the busbar protected zone.

An analysis of the simulation results in Table 8 shows that when an internal fault occurs the ratio *λ* in the presence of noise interference is greater than the ratio without the presence of noise interference. However, even if the noise is at 30 dB, the criterion in Eq (14) can still be satisfied and identified as an internal fault. When an external fault occurs, the ratio *λ* in the presence of noise interference is smaller than the ratio without the presence of noise interference. However, even if the noise is at 30 dB, the fault zone can still be reliably detected. Therefore, the criteria proposed in this paper are essentially not susceptible to noise and can reliably identify busbar fault zones.

**Table 8:** Test results of the protection algorithm in the case of different signal-to-noise ratios.

Fault location	SNR /(dB)	M_s_	N_s_	λ	Test result
ABC three phase short circuit occurs on busbar M; transitional resistance is 200Ω; fault inception angle is 60°	30	0.0047	0.0021	2.24	Internal
	35	0.0078	0.0031	2.52	Internal
	50	0.052	0.022	2.36	Internal
	70	0.36	0.17	2.12	Internal
A phase-to-ground short circuit occurs on L_4_ at a distance of 50km from busbar M; transitional resistance is 150Ω; fault Initial angle is 90°	30	0.024	0.00071	33.80	External
	35	0.035	0.00073	47.95	External
	50	0.061	0.00069	88.41	External
	70	0.54	0.00069	782.61	External

### Analysis of the data loss of sampling points

In actual engineering measurements, there are cases involving data loss. When an internal fault occurs in the busbar, no backward travelling wave can be detected from each transmission line during the time period [*t*_0_, *t*_0_ + 2*d*_min_/*v*], and the magnitude of the backward travelling wave is zero. Therefore, the loss of sampling points has no impact on the similarity *sim*. That is, when an internal fault occurs in the busbar, the similarity *sim* is essentially not susceptible to the loss of sampling points. When an external fault occurs on the busbar, only the backward traveling wave can be detected from the fault line during the time period [*t*_0_, *t*_0_ + 2*d*_min_/*v*], and no backward traveling wave can be detected from the nonfault line. Therefore, it is sufficient to only 4consider the loss of the sampling points of the fault line when an external fault occurs outside the busbar zone. Table 9 shows the simulation verification in the case of a loss of sampling points when a fault occurs on L_4_. Considering more serious cases, the missing sample points contain peak points and do not contain points whose data is zero.

**Table 9:** Results from a comparison of missing sample points when internal and external faults occurs on the busbar.

Fault location	Number of the sampling points being dropped	M_s_	N_s_	λ	Test result
BC phase short circuit fault occurs on L_4_ at a distance of 120km from busbar M; transitional resistance is 150Ω; fault Initial angle is 60°	10	1.00	0.0029	344.83	External
	30	1.00	0.0045	222.22	External
	50	1.00	0.0090	111.11	External
	No loss of sampling points	1.00	0.0025	400.00	External

An analysis of the simulation experiment of Table 9 shows that when an internal fault occurs no backward travelling wave can be detected from each transmission line during the time period [*t*_0_, *t*_0_ + 2*d*_min_/*v*], the Euclidean distance is approximately zero. At this time, loss of sample points cannot result in a misjudgment of the criterion. When an external fault occurs, only the backward traveling wave can be detected from the fault line during the time period [*t*_0_, *t*_0_ + 2*d*_min_/*v*] after the fault occurs, and the similarity between the transmission lines increases due to a data loss of the sampling points. However, even if 50 sampling points are lost, the criterion in Eq (14) can still be satisfied. Therefore, the performance of the protection algorithm is essentially not susceptible to data loss of the sampling points.

### Analysis of the operation speed

The current busbar protection widely applied in actual power systems is a current differential protection based on power frequency. The busbar differential protection mainly uses the Kirchhoff current theorem to identify whether the busbar is faulty. The principle of the current differential busbar protection is that when an external fault occurs on the busbar under normal operation the sum of the currents of the outgoing lines connected to the busbar is 0. When a fault occurs on the busbar, the currents of all the outgoing lines and the transformer branch connected to the busbar are equal to the total current of the fault point. Current differential protection uses a full-circumference or half-cycle Fourier algorithm for phasor calculations, the speed of which depends on the amount of computation of the algorithm and the required data window length. In terms of the amount of computation, applying a full-cycle Fourier algorithm to compute a phasor requires 2N multiplications and additions when sampling N points per power frequency cycle, while the half-cycle Fourier algorithm requires N multiplications and additions. Considering a sampling rate of 1600 Hz (32-point sampling), calculating a phasor with the implementation of a full-cycle Fourier algorithm requires 64 multiplications and additions, and calculating a phasor with the implementation of a half-cycle Fourier algorithm requires 32 multiplication algorithms. In terms of the data window length, in order to ensure the accuracy of calculation the full-cycle Fourier algorithm requires a data window of 20 ms, while the half-cycle Fourier algorithm requires a data window of 10 ms.

The amount of computation of the busbar protection algorithm flow (shown in Fig 9) is mainly embodied in the Clarke phase mode transformation, the S-transform and the distance calculation. After a rough estimation, the phase-mode transformation requires 18 multiplications. The amount of computation of the S-transform is usually large, and for the N-point discrete signal, the amount of computation required to complete the S-transform is approximately an *N*^2^ log_2_ *N* + *N*^2^ multiplication of the real numbers. The original signal length selected by the algorithm in this chapter is 100 (0.5 ms data window, 200 kHz sampling frequency), and the S-transform is implemented, which requires approximately 76,439 multiplications. At the same time, the distance calculation requires approximately 100 multiplications. Therefore, the algorithm requires approximately 76,557 multiplications and a small number of accumulation operations. A fast digital signal processing chip (DSP) can quickly complete the above operation. Using the DS1003 based on the TMS320C40 as an example, the above operation will not exceed 6 ms. If a higher dominant frequency DSP processor is used, the operation speed will be faster.

Although the computational complexity of the proposed method has reached a high level, the operation can be completed in 6 ms with a DSP, and the required data window length is only 0.5 ms. The time required for the algorithm to complete fault identification is approximately 6.5 ms, which greatly shortens the data window length compared with the conventional power frequency variation directional component. Therefore, the speed of the proposed algorithm will be much faster than that of the power frequency variation directional component. Table 10 compares the response times of the traditional current differential protection scheme and the proposed algorithm, where Ta is the protection response time.

**Table 10:** Comparison of protection response times of the busbar protection algorithm.

The current differential protection		The algorithm proposed in this paper
The full-cycle Fourier algorithm	The half-cycle Fourier algorithm	
Ta is greater than 20 ms	Ta is greater than 10 ms	Ta is less than 7 ms

## Conclusion

Based on the Euclidean distance algorithm, a busbar protection principle is proposed in this paper. The Euclidean distance is then used to analyze the similarity of waveforms of the backward traveling wave when internal and external faults occur on the busbar. The feasibility of the busbar protection criterion is verified by a simulation example. The theoretical analysis and simulation results show that:

1. An S-transform is applied in the algorithm to obtain a single-frequency initial backward traveling wave waveform. By analyzing the similarity of the waveforms of the S-transformed travelling waves of each related transmission line, the internal and external faults that occurred on the busbar are detected. The algorithm essentially overcomes the impact of such interference factors as the transitional resistance and the fault inception angle. The algorithm can correctly identify the internal and external faults that occurred on the busbar with a strong ability to resist interference.
2. The protection algorithm only uses the information of the first backward traveling wave, with a simple criterion, adjustable parameters settings, a short data window and small data transmission.
3. The algorithm is a combination of the directional traveling wave principle, the S-transform and the Euclidean distance algorithm. It has the characteristics of anti-TA saturation and is fairly responsive, highly sensitive, and quite practical.
